# Supplementary material for: Gini coefficients for measuring the distribution of sexually transmitted infections among individuals with different levels of sexual activity
Source: PeerJ. 2020 Jan 20;8:e8434. doi: 10.7717/peerj.8434 (PMC6977500; doi:10.7717/peerj.8434)
Supplement: Supplemental Information 1 [file peerj-08-8434-s001.pdf]

Gini coefficients for measuring the distribution of sexually transmitted infections among individuals with different sexual activity

Sandro Gsteiger[1], Nicola Low[1], Pam Sonnenberg[2], Catherine H Mercer[2], Christian L Althaus[1]

[1]Institute of Social and Preventive Medicine (ISPM), University of Bern, Switzerland
[2]Institute for Global Health, University College London, London, UK

Corresponding author: christian.althaus@alumni.ethz.ch

Supplemental Information

Table S1 Estimated Gini coefficients for different sexually transmitted infections in men

Infection	Gini coefficient	95% confidence interval (CI)
Chlamydia trachomatis	0.36	0.16 - 0.57
Mycoplasma genitalium	0.38	0.11 - 0.61
HPV 6	0.04	-0.24 - 0.38
HPV 11	-0.22	-0.31 - 0.10
HPV 16	0.19	-0.01 - 0.41
HPV 18	-0.06	-0.28 - 0.30

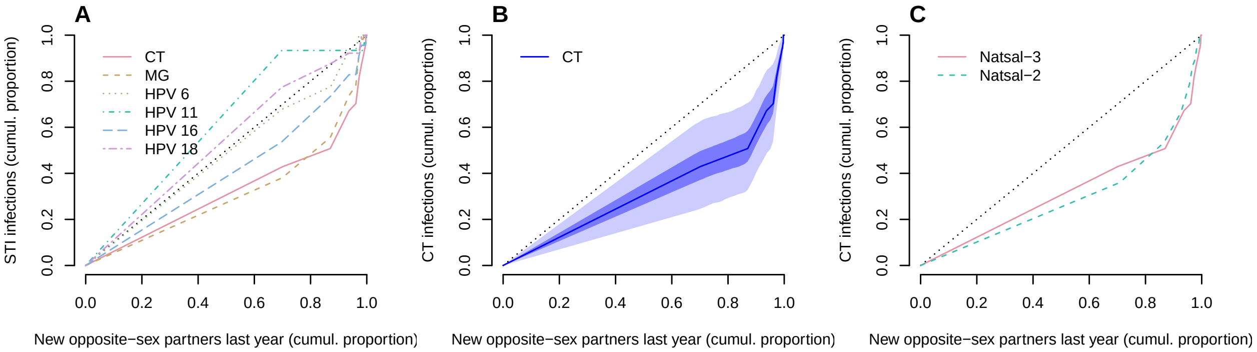

Figure S1 Lorenz curves representing the cumulative proportion of STI infections in men as a function of the cumulative proportion of the population, after population sub-groups have been ranked by the number of new opposite-sex partners in the last year. (A) Lorenz curves for different STIs. Data: Natsal-3. (B) Uncertainty around Lorenz curve for CT. The grey areas represent point-wise 50% (dark grey) and 95% (light grey) confidence bands. Data: Natsal-3. (C) Comparison of Lorenz curves for CT between Natsal-2 (dashed line) and Natsal-3 (solid line). In all graphs, the diagonal line (black dotted line) denotes perfect equality, i.e., an equal dispersion of the infection across population sub-groups.

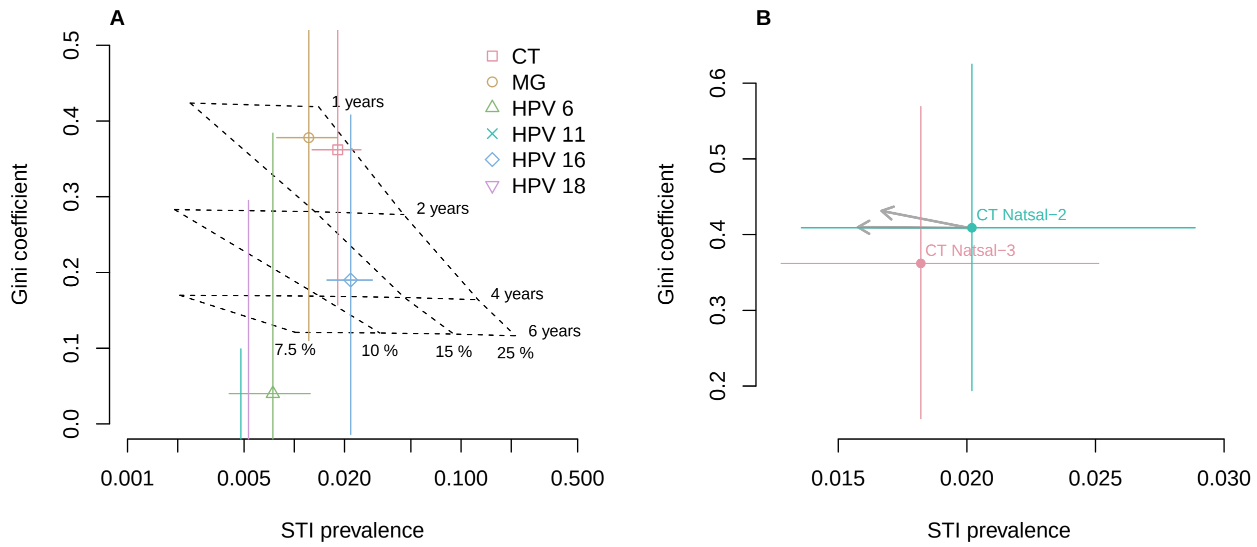

Figure S2 Relationship between Gini coefficient, STI prevalence, infectious duration and transmissibility. (A) Gini coefficients and STI prevalence for men in Natsal-3 (coloured dots). Modelled values for different combinations of the infectious duration and the per partnership transmission probability are projected on the graph (dashed grid). (B) Expected impact of control measures on Gini coefficient and prevalence of male CT between Natsal-2 and Natsal-3. The black arrows denote a 10% reduction in the per partnership transmission probability (horizontal arrows) or the infectious duration (diagonal arrows).
